# Comparison of the market share of public and private hospitals under different Medical Alliances: an interrupted time-series analysis in rural China

**DOI:** 10.1186/s12913-024-10941-0

**Published:** 2024-04-22

**Authors:** Yingbei Xiong, Kunhe Lin, Yifan Yao, Zhengdong Zhong, Li Xiang

**Affiliations:** 1 Department of Health Management, School of Medicine and Health Management, Tongji Medical College, Huazhong University of Science and Technology, Hangkong Road 13, 430030 Wuhan, China; 2 HUST base of National Institute of healthcare Security, Wuhan, China

**Keywords:** Health equity, Medical alliances (MAs), Public-private partnership, Market share

## Abstract

**Background:**

China initiated the Medical Alliances (MAs) reform to enhance resource allocation efficiency and ensure equitable healthcare. In response to challenges posed by the predominance of public hospitals, the reform explores public-private partnerships within the MAs. Notably, private hospitals can now participate as either leading or member institutions. This study aims to evaluate the dynamic shifts in market share between public and private hospitals across diverse MAs models.

**Methods:**

Data spanning April 2017 to March 2019 for Dangyang County’s MA and January 2018 to December 2019 for Qianjiang County’s MA were analyzed. Interrupted periods occurred in April 2018 and January 2019. Using independent sample t-tests, chi-square tests, and interrupted time series analysis (ITSA), we compared the proportion of hospital revenue, the proportion of visits for treatment, and the average hospitalization days of discharged patients between leading public hospitals and leading private hospitals, as well as between member public hospitals and member private hospitals before and after the reform.

**Results:**

After the MAs reform, the revenue proportion decreased for leading public and private hospitals, while member hospitals saw an increase. However, ITSA revealed a notable rise trend in revenue proportion for leading private hospitals (*p* < 0.001), with a slope of 0.279% per month. Member public and private hospitals experienced decreasing revenue proportions, with outpatient visits proportions declining in member public hospitals by 0.089% per month (*p* < 0.05) and inpatient admissions proportions dropping in member private hospitals by 0.752% per month (*p* < 0.001). The average length of stay in member private hospitals increased by 0.321 days per month after the reform (*p* < 0.01).

**Conclusions:**

This study underscores the imperative to reinforce oversight and constraints on leading hospitals, especially private leading hospitals, to curb the trend of diverting patients from member hospitals. At the same time, for private hospitals that are at a disadvantage in competition and may lead to unreasonable prolongation of hospital stay, this kind of behavior can be avoided by strengthening supervision or granting leadership.

## Background

In the context of constrained medical resources, optimizing resource allocation to enhance service efficiency and ensure equity is a global imperative [1, 2]. International experiences have pointed out the effectiveness of an integrated healthcare system in solving the above problems [3]. The reform of Medical Alliances (MAs) stands out as a significant exploration and practical model within this system [4]. By vertically integrating all levels of the hospitals within the county, the MAs reform intends to achieve medical resource integration, and promote the development of primary hospitals with weaker service capacity through providing high-quality medical resources and technical guidance from high-level hospitals, and ultimately minimizes health inequality across the county. The primary hospitals commonly refer to township hospitals with fewer than 100 beds. Their responsibilities include offering preventive care, basic healthcare services, and rehabilitation services. China implemented the MAs reform in 2009. In December 2023, with the release of *Guiding Opinions on Comprehensively Promoting the Construction of Tight Medical Alliances*, the MAs reform has become one of the most important development directions in China’s medical industry [5].

Typically, MAs include two distinct hospital types. The first is the leading hospital. There is only one leading hospital in a MA, characterized by its strongest medical service capabilities and vested authority to plan, decide, manage, and distribute benefits across the entire MAs. The second comprises member hospitals, which are composed of hospitals with weaker service capacity, and mainly provide medical services without managerial authority over the MAs. Leading hospitals assume a pivotal role by offering guidance and support to member hospitals. The leading and member hospitals of a MA are designated by the local government.

In China, hospitals are generally divided into public and private types according to their ownership. The growing significance of private healthcare providers has spurred heightened attention to the management of public-private partnerships [6]. Effectively managing this model is a prominent subject of discourse [7–11]. Previous studies have demonstrated that by integrating public and private sector members, MAs can significantly improve access and equity, while reducing the drawbacks of public hospital monopolies [12–14]. China is also actively promoting private hospitals to join the MAs [15]. In June 2019, China released a blockbuster policy, *Notice on Printing and Issuing Opinions on Promoting the Sustainable, Healthy and Standardized Development of Private Hospitals*, stated that private hospitals can choose to join the MAs, and those with strong comprehensive strength or specialist service capabilities can lead the formation of MAs and encourage moderate competition inside. At present, there are two main forms of private hospitals participating in MAs in China, one is as a member hospital, and the other is as a leading hospital. By 2020, a total of 7,840 private hospitals have become integral participants in the MAs, accounting for 33.3% of the total number of 23,524 private hospitals in China in the year.

However, at present, the healthcare landscape in the majority of developing nations is characterized by the predominance of public hospitals, with a minimal market share held by private hospitals. Notably, hospitalization services and surgical treatment of complex cases are mainly provided by public hospitals [16–19]. In the case of unbalanced resource allocation and the system dominated by public hospitals, the participation of private hospitals in MAs has defects in resources, capabilities and reputation, and also faces obstacles in concepts, interests and systems. The absence of established theoretical frameworks and mature practice models further compounds the challenges faced by private hospitals seeking integration into the MAs. Although the entry of private hospitals into the MAs may improve the overall service level of the MAs, the impact on public hospitals and private hospitals themselves remain ambiguous and warrant comprehensive investigation.

The majority of extant studies have primarily explored disparities in service provision between public and private hospitals [20–23].Some studies have shown that the response to policy interventions differs between public and private hospitals [24–27]. In addition, the current research on MAs mainly focused on the qualitative analysis of the policy content [5] and the implementation effect of medical reform [28], as well as the analysis of the impact of MAs on the service quality of medical institutions [29] and patient service utilization [30]. However, the existing research mostly focused on the conventional MAs dominated by public hospitals [31], little attention has been paid to the two forms of incorporating private hospitals into MAs promoted in China. Additionally, there was no research on a different impact on public and private hospitals under MAs reform. Moreover, in the context of public-private partnerships within MAs, an aspect that warrants consideration is the delineation of leadership responsibilities between public and private medical institution [32, 33].

In April 2018, Dangyang County launched the MAs reform. The leading hospital is the public municipal people’s hospital, and there were one public hospital and three private hospitals as the member hospitals. In January 2019, Qianjiang County carried out the MAs reform, and the leading hospital of MA was a private hospital, including several public primary hospitals as the member hospitals. Therefore, focusing on the diverse forms of private hospital engagement within MAs, we analyzed two distinct MA models: one with public hospitals performing leadership roles having member private hospitals in their alliance; the other where private hospitals take the lead with public hospitals as members. The primary objective was to compare the changes in market shares between public and private hospitals under these two MA models. Furthermore, we aimed to explicate the underlying factors contributing to these observed variations.

## Methods

### Study design and data sources

As shown in Table 1, the study design was based on a retrospective comparative study. We selected two counties from Hubei Province, Dangyang County and Qianjiang County, as the sample area of this study. Hubei Province is located in the central region of China. In 2021, the per capita GDP of Dangyang County was approximately USD 21,157.5, while in Qianjiang County, it was USD 15,810. Comparatively, the per capita GDP among the top 100 counties and cities for the same period averaged USD 17,794. In 2021, the number of beds per thousand people was 6.16 and health professionals per thousand people was 5.88 in Danyang. In Qianjiang County during the same year, these figures were 6.18 and 6.32, respectively. When considering the entire cohort of counties in China, the corresponding averages were 6.01 beds and 6.27 health professionals per thousand people. In Dangyang County, the average life expectancy until 2025 was 79 years old, the sex ratio male/female was 1.02, the per capita disposable income of permanent urban residents was USD 6,311.14, the per capita disposable income of permanent rural residents was USD 4,125.33, and the proportion of education expenditure in local general public budget expenditure was 13.26%. In Qianjiang County during the same year, these figures were 80.5 years old, 1.03, USD 5,732.68, USD 3,277.63 and 17.40%. The economic and social development, health resources level and demographic index level of the two counties were comparable, and they were at the national average level. Their MA models have substantial reference value for comparable counties regarding socioeconomic and health services aspects.

**Table 1:** Basic situation of the two sample areas

	Dangyang County	Qianjiang County	National level
Per capita GDP (USD)	21,157.5	15,810	17,794
The number of beds per thousand people in medical and health institutions	6.16	6.18	6.01
The number of health professionals per thousand people in medical and health institutions	5.88	6.32	6.27
Average life expectancy until 2025 (year)	79.0	80.5	78.3
Sex ratio male/female	1.02	1.03	1.05
Per capita disposable income of permanent urban residents (USD)	6,311.14	5,732.68	7,348.86
Per capita disposable income of permanent rural residents (USD)	4,125.33	3,277.63	2,934.31
The proportion of education expenditure in local general public budget expenditure (%)	13.26	17.40	16.99

As shown in Table 2, in April 2018 and January 2019, Dangyang County and Qianjiang County, under government auspices, established their own MAs respectively. In the case of Dangyang County, the MA comprises two public hospitals, three private hospitals, and several public primary hospitals. One of the public hospitals assumes the role of the leading hospital within this MA. For the second sample area, the leading hospital of MA in Qianjiang County was a private hospital, with seven public primary hospitals as the member hospitals. We collected data from one leading public hospital, one member public hospital and three member private hospitals in Dangyang County, and also one leading private hospital in Qianjiang County for comparison with the leading public hospital in Dangyang County. In Dangyang County’s MA, the number of beds of MA’s hospitals was 1,629, the number of doctors of MA’s hospitals was 454, the number of discharged patients of MA’s hospitals was 47,522, the total medical income of MA’s hospitals was USD 81,690,192.50 in 2021. These figures were 820, 390, 25,680 and USD 74,729,840.00 in Qianjiang County’s MA at the same year.

**Table 2:** Basic situation of the two sample MAs

	Dangyang County	Qianjiang County
The onset of the MAs	Apr-18	Jan-19
Leading hospital	1 public hospital	1 private hospital
Member hospital	1 public hospitals 3 private hospitals 10 public primary hospitals	7 public primary hospitals
The number of beds of MAs’ hospitals	1,629	820
The number of doctors of MAs’ hospitals	454	390
The number of discharged patients of MAs’ hospitals	47,522	25,680
Total medical income of MAs’ hospitals (USD)	81,690,192.50	74,729,840.00

This research was undertaken as a time series analysis spanning a 24-month duration, with data sourced from the Health Commission information system. Data collection encompassed the period from April 2017 to March 2019 in Dangyang and from January 2018 to December 2019 in Qianjiang. April 2018 (the onset of the MAs reform in Dangyang) and January 2019 (the onset of the MAs reform in Qianjiang) were considered as interrupted time.

### Variables and outcomes

In this research, we have selected outcome variables across three key dimensions: the proportion of hospital revenue, the proportion of visits for treatment, and the average hospitalization days of discharged patients. The proportion of visits for treatment includes the proportion of total outpatient visits and the proportion of inpatient visits. We use the proportions to determine the hospital market share, which was calculated by dividing the absolute value of the hospital revenue or service volume by the absolute value of the hospital revenue or service volume in that county. In addition to the impact of policy intervention, the service volume and revenue level of hospitals are also affected by natural growth factors [34]. Relative numbers can well eliminate the errors caused by the resource advantages and patient preferences implied by the absolute value, and objectively compare the changes in the market share of different medical institutions in different counties. In previous studies, many scholars have also used relative numbers to compare the market shares of different medical institutions [35, 36], understand the size and pattern of the private sector in mixed health systems in different countries [7]. The average hospitalization days of discharged patients is used to reflect the efficiency of medical services in medical institutions, and to analyze whether there is a way to produce unnecessary medical expenses by extending the average length of stay (Table 3).

**Table 3:** The variables used in this study

Variable	Definition	Calculation formula
The proportion of hospital revenue (%)	The proportion of the hospital’s revenue to the total revenue of all medical institutions in the county	The hospital’s revenue/ total revenue of all medical institutions in the county
The proportion of total outpatient visits (%)	The proportion of the hospital’s outpatient visits to the total outpatient visits of all medical institutions in the county	The hospital’s outpatient visits/ total outpatient visits of all medical institutions in the county
The proportion of total inpatient visits (%)	The proportion of the hospital’s inpatient visits to the total inpatient visits of all medical institutions in the county	The hospital’s inpatient visits/ total inpatient visits of all medical institutions in the county
Average hospitalization days of discharged patients (days)	The average length of hospital stays for each patient in a certain period	Total length of hospital stays for discharged patients/ Number of discharged patients

### Statistical analyses

#### Independent samples t-tests and chi-square tests

Independent sample t-test was used to compare the average hospitalization days of discharged patients before and after the reform of sample medical institutions. Chi-square test was used to compare the proportion of hospital revenue, the proportion of visits for treatment before and after the reform. All analyses were performed using all available data in SPSS version 26. *p* < 0.05 was considered statistically significant.

#### Interrupted time-series analysis

Interrupted Time Series Analysis (ITSA) is increasingly being used in the field of health policy. It is especially used for interventions or events in a certain time period and determining their health outcomes impact [37, 38]. ITSA has been used for the assessment of a different type of interventions or event including health system reform [39], healthcare price reform, medical security policy reform [40], and so on. It was also used for the impact evaluation of MAs [30].

The two key variables in the model of ITSA include the level and trend variable, which are used to indicate the impact of the intervention. The immediate and long-term effects of the interventions, are derived from the level and trend variable, respectively. The following regression model shows a simple-group ITSA [41].

*Y*_t_ = *β*_0_ + *β*_1_*T*_t_ + *β*_2_*X*_t_ + *β*_3_*T*_t_*X*_t_+ ϵt

In this model, *Y*_t_ represents the outcome variable, including three dimensions. *T*_t_ represents the time trend, indicating the number of months from the beginning of observation period to time t. *X*_t_ represents the MAs reform intervention, which is assigned to 0 before the intervention and equals to 1 after that. *T*_t_*X*_t_ is the interactive effects of time and intervention. The error term ϵt represents the random variability that cannot be explained by the model. *β*_0_, *β*_1_, *β*_2_ and *β*_3_ show the intercept level before the reform, monthly trend before the reform, step change at the reform (immediate effect), and monthly trend change after reform (long-term effect), respectively. Through the ITSA, the baseline level and trend can be effectively controlled, and the influence of other intervention factors other than the policy reform concerned in this study can be exclude, so that the level and trend changes caused by the reform can be analyzed. In this study, Durbin-Watson was used to test whether there was autocorrelation, and generalized least square method was used to correct if there was. Dicky-Fuller test is used to determine whether it has seasonality, and seasonal correction is performed if there is seasonality.

## Results

### Descriptive analysis

Table 4 shows that, the proportion of hospital revenue of leading public and private hospitals was 63.42% and 37.90% before the reform, and decreased to 60.16% and 34.93% after the reform, respectively. In the meanwhile, the proportion of member public and private hospitals all increased after the reform (*p*< 0.001). In addition to the leading public hospital, the proportion of outpatient visits in the other three types of hospitals all showed varying degrees of reduction (*p* < 0.001). The proportion of inpatient visits in leading public and private hospitals increased, while that in member public hospitals declined (*p* < 0.001). As for the average hospitalization days of discharged patients, both the leading and member public hospitals experienced a significant decline after the reform (*p* < 0.05).

**Table 4:** Independent samples t-tests and chi-square tests results

	Pre-reform	Post-reform	statistics	*P* Value
**The proportion of hospital revenue (%)**				
Leading public hospital	63.42	60.16	999.05	0.000
Leading private hospital	37.90	34.93	2068.53	0.000
Member public hospital	16.66	18.45	488.75	0.000
Member private hospital	19.39	20.53	180.57	0.000
**The proportion of total outpatient visits (%)**				
Leading public hospital	60.36	63.40	1257.03	0.000
Leading private hospital	35.73	32.88	2670.70	0.000
Member public hospital	20.36	19.38	193.27	0.000
Member private hospital	18.20	16.73	482.72	0.000
**The proportion of total inpatient visits (%)**				
Leading public hospital	60.91	58.07	89.66	0.000
Leading private hospital	25.50	25.07	5.34	0.021
Member public hospital	13.35	16.06	157.24	0.000
Member private hospital	25.86	26.05	0.51	0.474
**Average hospitalization days of discharged patients (days)**				
Leading public hospital	9.13 ± 0.55	8.58 ± 0.30	0.121	0.006
Leading private hospital	10.43 ± 0.44	10.22 ± 0.24	1.408	0.173
Member public hospital	10.87 ± 1.01	9.84 ± 0.57	3.061	0.006
Member private hospital	15.29 ± 11.60	16.16 ± 17.31	-0.252	0.801

### Interrupted time-series analysis

#### Effect on the proportion of hospital revenue

**Fig. 1:**
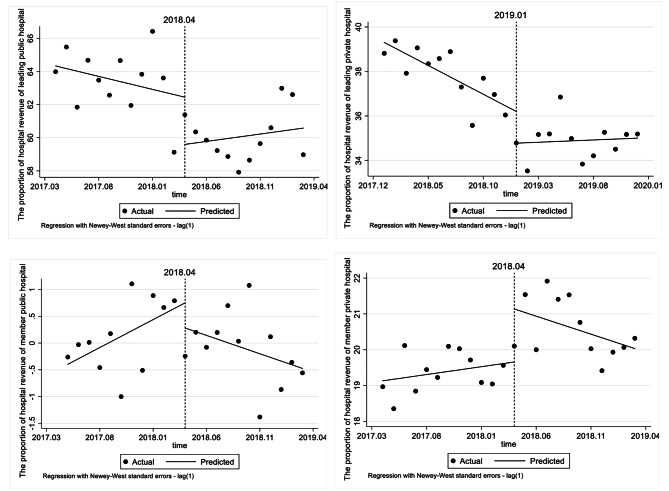
ITSA results of the effect on the proportion of hospital revenue

Tables 5 and Fig. 1 shows that, the proportion of the leading private hospital revenue deescalated by 1.421% at the moment of the reform (*p* < 0.05) and increased by 0.279% after that (*p* < 0.001). The proportion of hospital revenue of member public hospital showed a significant upward trend by 0.104% before the reform, and decreased sharply by 0.173% after the reform (*p* < 0.001). In the meanwhile, the proportion of hospital revenue of member private hospital increased by 1.480% at the moment of the reform, and decreased by 0.144% after the reform (*p* < 0.05).

**Table 5:** ITSA results of the effect on the proportion of hospital revenue

	Constant β1 (CI)	SE	*P* value	Monthly trend before reform β2(CI)	SE	*P* value	Step change at the reform β3(CI)	SE	*P* value	Change in trend after reform β4(CI)	SE	*P* value
Leading public hospital	64.333	0.631	0.000^(***)^	-0.157	0.181	0.396	-2.851	2.041	0.178	0.247	0.214	0.262
Leading private hospital	39.304	0.232	0.000^(***)^	-0.259	0.041	0.000^(***)^	-1.421	0.640	0.038^(*)^	0.279	0.066	0.000^(***)^
Member public hospital	-0.397	0.167	0.028^(*)^	0.104	0.026	0.001^(**)^	-0.472	0.313	0.148	-0.173	0.036	0.000^(***)^
Member private hospital	19.132	0.246	0.000^(***)^	0.044	0.040	0.287	1.480	0.504	0.008^(**)^	-0.144	0.065	0.039^(*)^

#### Effect on the proportion of total outpatient visits

**Fig. 2:**
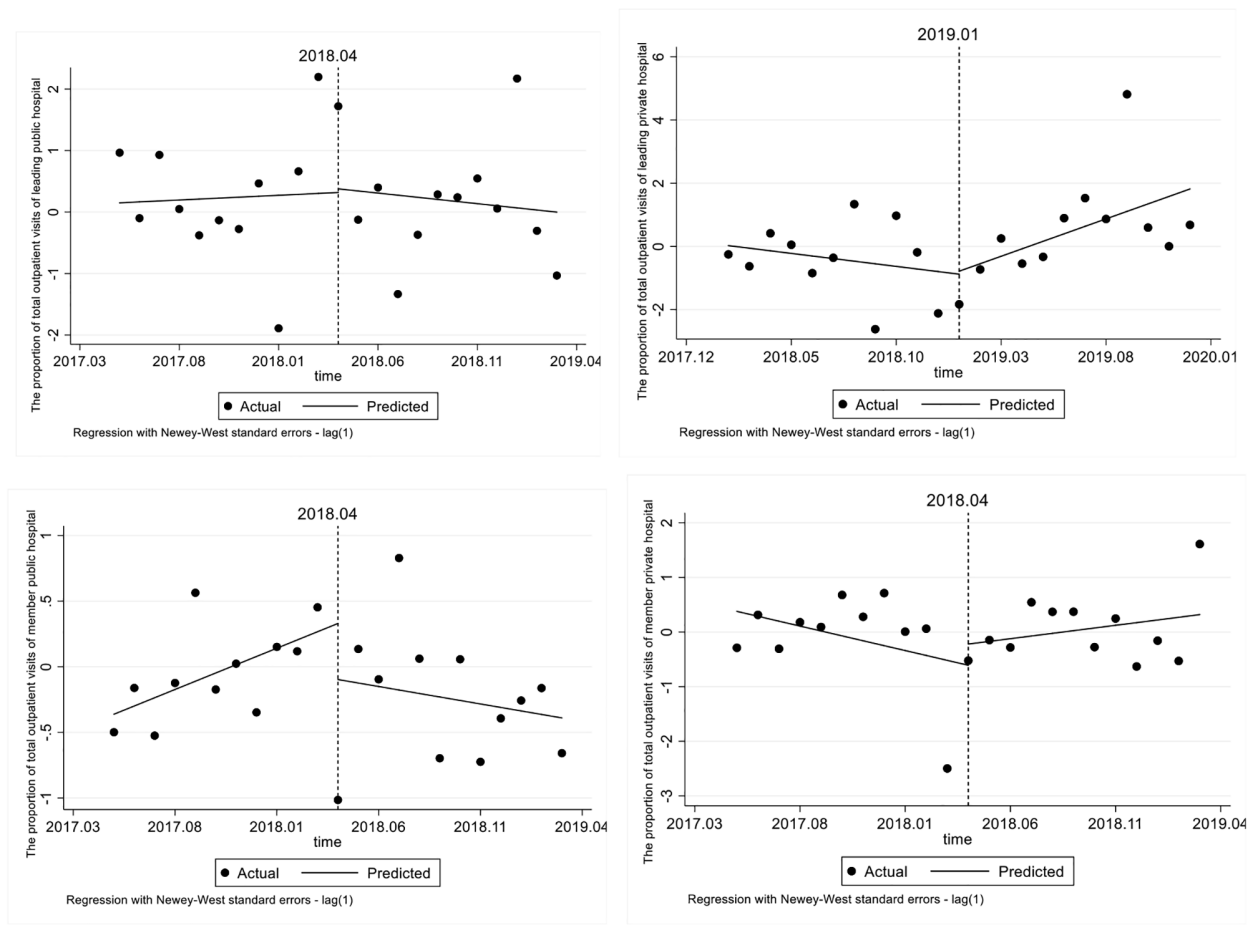
ITSA results of the effect on the proportion of total outpatient visits

As shown in Tables 6 and Fig. 2, the proportion of total outpatient visits of the leading public hospital has no significantly change after the reform (*β*4 = − 0.050), but the trend contrasted with that of the leading private hospital (*β*4 = 0.319). The proportion of total outpatient visits of member public hospital has shown an increasing trend by 0.063% before the reform (*p* < 0.01), and decreased by 0.089% per month (*p* < 0.05) after the reform, while the change of member private hospital showed no significant change.

**Table 6:** ITSA results of the effect on the proportion of total outpatient visits

	Constant β1 (CI)	SE	*P* value	Monthly trend before introduction β2(CI)	SE	*P* value	Step change when introduced β3(CI)	SE	*P* value	Change in trend after introduction β4(CI)	SE	*P* value
Leading public hospital	0.150	0.445	0.740	0.015	0.123	0.903	0.059	0.985	0.953	-0.050	0.171	0.775
Leading private hospital	0.027	0.338	0.938	-0.082	0.084	0.342	0.094	0.731	0.899	0.319	0.160	0.061
Member public hospital	-0.362	0.120	0.007^(**)^	0.063	0.016	0.001^(**)^	-0.427	0.403	0.302	-0.089	0.042	0.046^(*)^
Member private hospital	0.378	0.390	0.345	-0.090	0.102	0.393	0.388	0.805	0.635	0.139	0.123	0.274

### Effect on the proportion of total inpatient visits

**Fig. 3:**
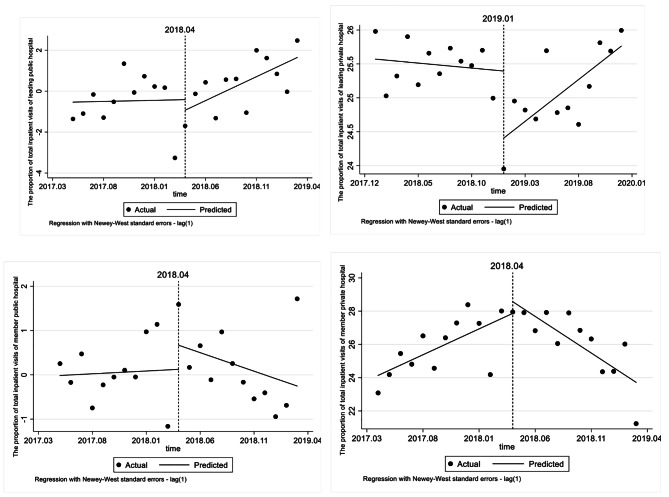
ITSA results of the effect on the proportion of total inpatient visits

Tables 7 and Fig. 3 shows the change trend of the proportion of total inpatient visits, the leading private hospital sharply decreased by 0.989% at the moment of reform, and suffered an elevation by 0.138% after the reform (*p* < 0.01). The proportion of total inpatient visits in member private hospital increased by 0.311% per month before the reform (*p* < 0.05), and decreased by 0.752% per month after the reform (*p* < 0.001). The proportion of inpatient visits in public hospitals did not change significantly.

**Table 7:** ITSA results of the effect on the proportion of total inpatient visits

	Constant β1 (CI)	SE	*P* value	Monthly trend before introduction β2(CI)	SE	*P* value	Step change when introduced β3(CI)	SE	*P* value	Change in trend after introduction β4(CI)	SE	*P* value
Leading public hospital	-0.536	0.604	0.385	0.011	0.150	0.942	-0.516	1.123	0.651	0.223	0.178	0.225
Leading private hospital	25.571	0.181	0.000^(***)^	-0.015	0.027	0.588	-0.989	0.208	0.000^(***)^	0.138	0.042	0.004^(**)^
Member public hospital	-0.015	0.249	0.953	0.013	0.072	0.863	0.544	0.804	0.506	-0.096	0.105	0.368
Member private hospital	24.135	0.490	0.000^(***)^	0.311	0.115	0.014^(*)^	0.701	1.137	0.544	-0.752	0.150	0.000^(***)^

### Effect on the average hospitalization days of discharged patients

**Fig. 4:**
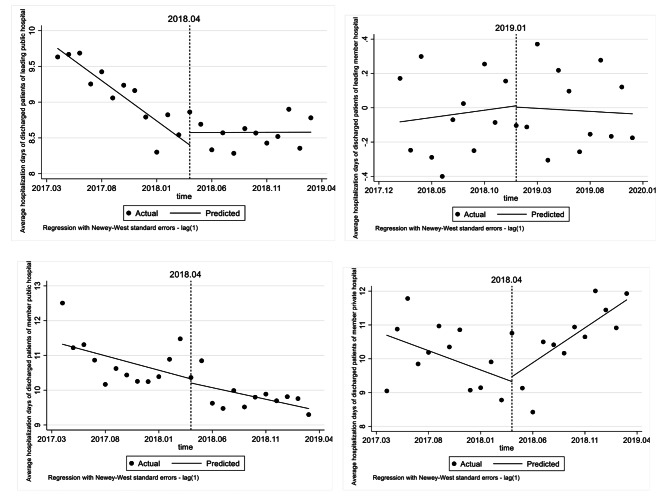
ITSA results of the effect on the average hospitalization days of discharged patients

Tables 8 and Fig. 4 shows that, the average hospitalization days in leading public hospitals has gradually stabilized from the sharp decline before the reform (*p* < 0.001, *β*2 = − 0.113, *β*4 = 0.113). The member private hospital has exhibited an upward trend in the average hospitalization days by 0.321 days per month (*p* < 0.01).

**Table 8:** ITSA results of the effect on the average hospitalization days of discharged patients

	Constant β1 (CI)	SE	*P* value	Monthly trend before introduction β2(CI)	SE	*P* value	Step change when introduced β3(CI)	SE	*P* value	Change in trend after introduction β4(CI)	SE	*P* value
Leading public hospital	9.751	0.056	0.000^(***)^	-0.113	0.013	0.000^(***)^	0.174	0.174	0.330	0.113	0.022	0.000^(***)^
Leading private hospital	-0.082	0.116	0.484	0.009	0.015	0.580	-0.008	0.126	0.952	-0.012	0.018	0.503
Member public hospital	11.322	0.452	0.000^(***)^	-0.083	0.075	0.280	-0.118	0.582	0.841	0.016	0.083	0.847
Member private hospital	10.697	0.608	0.000^(***)^	-0.114	0.081	0.174	0.144	0.770	0.853	0.321	0.100	0.004^(**)^

## Discussion

The revenue proportion of leading public hospitals decreased from 63.42 to 60.16%, and that of leading private hospitals decreased from 37.90 to 34.93%. Concurrently, there was an observed increase in the proportion of member hospitals, aligning with patterns identified in previous research [42]. It indicated that the MAs reform has effectively improved the capacity of member hospitals through sinking leading hospitals’ resources, which further guaranteed the balanced distribution of high-quality resources and the fairness of services.

### Public hospital maintained steady revenue proportions, whereas private hospitals displayed fluctuating and ascending proportions when both serving as leading institutions

From the results of ITSA, when both public and private hospitals were the leading hospitals, the revenue proportion of public hospital was not significantly affected. In contrast, the revenue proportion of private hospital experienced a decline during the reform period, followed by a subsequent rebound. Previous studies have shown the generally higher medical quality and service efficiency of public hospitals compared to private hospitals [43, 44]. This inherent competitiveness allows public hospitals to sustain a stable revenue proportion. Private hospitals were at a disadvantage in the competition with other public hospitals, which resulted in their instantaneous decrease trend. Meanwhile, private hospitals that can be selected as MAs leading hospitals have strong service capabilities themselves. Furthermore, the leaders of leading private hospitals will also have stronger enthusiasm due to the leadership of MAs [45]. In addition, leading hospitals can provide higher income and better career development opportunities, so more medical service providers with higher education level would be attracted to the leading private hospital [46]. Therefore, the leading private hospitals have certain recovery capability, the proportion of hospital revenue in the later stage of reform has rebounded significantly.

### Secondly, the revenue proportion of member hospitals with different ownerships showed a downward trend

When both public and private hospitals were member hospitals, the proportion of hospital revenue decreased after joining MAs. This phenomenon can be attributed to various factors. Firstly, when the leading hospital has a greater power to formulate performance evaluation standards and allocate surplus funds, it will drive it to extract patients from member hospitals through MAs to earn more medical funds [47, 48]. Additionally, the selection criteria employed by leading hospitals tend to favor institutions with robust medical service capabilities, featuring high-level medical professionals, advanced diagnostic and treatment technologies, and extensive resources such as large-scale equipment. In order to increase hospital revenue, the leading medical institutions may engage in overtreatment, which is a common problem in China [30, 49]. Moreover, even if the higher-level medical institutions have the willingness to refer patients downward, the constrained availability of resources such as equipment, beds, and drugs in member hospitals may limit the actualization of downward referrals [50]. Although the results of descriptive analysis indicated positive outcomes from the MAs reform, it was still necessary to strengthen the supervision of the leading hospitals, enhance the service capacity of member hospitals, in order to curb the trend of diverting patients from member hospitals.

### In addition, the reasons for the change of revenue proportion in member hospitals with were disparate between the two ownership types

Specifically, the proportion of outpatients in member public hospitals decreased after the reform, whereas in member private hospitals, it was mainly the proportion of inpatients that declined. Previous studies have also shown that the proportion of hospitalizations in public hospitals is generally higher than that of private hospitals, and most of them were complex cases or operations required to treat patients with multiple injuries [17–19]. On the one hand, due to the different functional positioning of inpatient services and outpatient services, it is more difficult to treat diseases that require hospitalization and the level of medical services varies greatly among different medical institutions. On the other hand, the level of hospitalization expenses in the private sector is higher than that in public hospitals [17]. Therefore, patients generally choose public medical institutions with more advanced medical technology [51, 52] and cheaper medical expenses for hospitalization in China, thereby contributing to the observed decline in inpatient proportions within member private hospitals. For outpatient services with relatively low technical complexity, patients will be more inclined to go to private hospitals when there is not much difference in the level of outpatient expenses between public and private hospitals. The superior patient experience often encountered in private healthcare facilities also influences this trend, thereby resulting in a reduction in the proportion of outpatient visits to public hospitals.

### Finally, empowering private hospitals with leadership roles or reinforcing oversight mechanisms may constrain their unreasonable behavior

According to the results of the average length of stay, the level of public hospitals has remained relatively stable. The post-reform level (8.58 days) of leading public hospitals was lower than the average level of China in 2021 (9.2 days). Conversely, the average length of stay of member private hospital increased by 0.321 days per month after the reform (*p* < 0.01). The leading private hospital did not appear the unreasonable behavior. As member hospitals, the private hospitals need to face the strong competition from both leading hospital and public hospitals at the same time. When they were unable to increase their proportion of hospitalizations, in response, they may choose unconventional efforts and higher risk strategies, such as extending hospitalization time, to make up for losses in hospital revenue [53]. Therefore, to provide health care through private providers requires strong regulatory, management and information capabilities [54].

### Limitations of the study

This study has several limitations. Firstly, in addition to the MAs reform, there may be other disruptive factors affecting the changes in the market share of public and private hospitals, such as medical technology and equipment, patient needs and preferences, geographical location and transportation accessibility, which may also have potential impacts. Secondly, our study utilized a 24-month period for the comparative analysis using ITSA, although this timeframe allowed us to examine market share changes, it may not capture longer-term trends or account for potential changes beyond the observed period. Future studies with longer observation periods could provide a more comprehensive understanding. Thirdly, the specific characteristics of the sample regions and hospitals may not be fully representative of the broader healthcare landscape. The applicability of our results to other regions or healthcare alliance systems should be interpreted with caution.

## Conclusion

In conclusion, considering the emerging trends of the MAs reform, it is essential to emphasize regulatory oversight and guidance for both public and private leading hospitals to curb the tendency of excessive resource concentration. This study provides new evidence demonstrating that the changes in market share and underlying factors differ between public and private hospitals under different MAs. Private hospitals have shown tendencies towards unreasonable profit-seeking behaviors when faced with competition. To address this, granting leadership authority to private hospitals and strengthening regulatory mechanisms may constrain such behaviors. These findings provided a strong practical basis for the optimization of MAs reform. The practical implications of our research underscore the necessity for nuanced policy adjustments that account for the distinct challenges faced by public and private hospitals, thereby contributing to a more equitable and efficient healthcare landscape.

## Data Availability

The data that support the findings of this study are available from Dangyang and Qianjiang Health Commission information system but restrictions apply to the availability of these data, which were used under license for the current study, and so are not publicly available. Data are however available from the corresponding author upon reasonable request and with permission of Dangyang and Qianjiang Health Commission.

## Abbreviations

MAs: Medical Alliances
ITSA: interrupted time series analysis
